# Challenging the Status Quo to Shape Food Systems Transformation from a Nutritional and Food Security Perspective: Second Edition

**DOI:** 10.3390/foods12091825

**Published:** 2023-04-28

**Authors:** António Raposo, Renata Puppin Zandonadi, Raquel Braz Assunção Botelho

**Affiliations:** 1 CBIOS (Research Center for Biosciences and Health Technologies), Universidade Lusófona de Humanidades e Tecnologias, Campo Grande 376, 1749-024 Lisboa, Portugal; 2 University of Brasília, Faculty of Health Sciences, Nutrition Department, Campus Universitário Darcy Ribeiro, Brasília 70910-900, Brazil; renatapz@unb.br (R.P.Z.); raquelbotelho@unb.br (R.B.A.B.)

Access to and choices of food are doubtless beyond the realms of biological and nutritional needs. This does not mean that limitations to individuals’ access to the social, cultural, economic, and psychological aspects should also be overlooked. Access to safe, nutritious, pleasant, sustainable, and healthy food for all individuals should be the focus of food producers, industries, services and policymakers [1,2,3,4]. In this sense, food security and nutrition (FSN) have been in the spotlight of the international development agenda for decades, with development priorities and challenges oscillating over the years. FSN plays a central role in global policy as one of the world’s targets most urgently in need of achieving, particularly in the current COVID-19 pandemic era, and has become a mission-critical goal [5]. In addition to the pandemic period, a conflict between two agricultural powers, Russia and Ukraine, has induced several negative socioeconomic impacts on global FSN since the war is producing direct and indirect cascading effects on food production [6]. In many countries, the FSN level represents the level of self-sufficiency and population well-being [5]. In the broader analysis, policies that guarantee food security (in all aspects) and reduce hunger and malnutrition work to promote strong economies, human and planetary health, and sustainable development. Therefore, it is imperative that we positively change food systems in order to ensure universal access to sustainable, safe, and healthy food. Overall, FSN is a complicated and multi-faceted issue that cannot be restricted to a single variable, necessitating the deeper integration of various multi-disciplinary interventions [5].

Considering these premises and the continuous demand for, interest in, and topicality of the subject, as demonstrated in the Special Issue first edition [7], we promoted this second edition in order to promote discussions of food access (affordability, allocation, and preference of food); food availability (the production, distribution, and exchange of food); circularity in food systems at local, regional, or global levels; development, impact, and ethics of novel and data-driven technologies in food systems; and food security and policy, governance, institutions, and trade. These are the factors influencing food consumption and demand considering the food environment; the stability and dynamics of food security aspects; sustainable food systems and agro-ecological food production; and the utilization of the nutritional value, social value, and safety of food.

Healthy and sustainable food systems are essential in the efforts to meet increasing food security, nutrition and health demands [8]. Despite the demand for healthy and sustainable food products, the priority food system components of food purchase and food service require transformation in order to protect the population and planetary health [8]. The ongoing environmental disruptions will increase the demands to develop healthy food products in order to be resilient to changing environmental circumstances and to have a low environmental footprint [8]. Interaction among food producers, food industry, food services and policymakers is imperative to the achievement of sustainable FSN [5,8]. The FSN drivers and policies should be used by policymakers to improve food security, contributing to sustainable food production and food policies for different income groups in order to promote a sustainable food system transformation [5,9].

There is great concern about animal products consumption and the impact of the practice on health and the environment. In this sense, the food industry and consumers are searching for alternatives to reduce animal product consumption globally. However, the use of plant-based foods as substitutes for animal products is challenging from technological, sensory, nutritional, consumer acceptance and sustainable points of view [10,11]. Further studies on animal product substitutes’ effect on environmental pollution reduction, safety, and ethical risk perception are required to construct an effective animal-product substitute regulatory system [10].
